# Socioeconomic inequalities in the relationship between internet usage patterns and depressive symptoms: Evidence from a Chinese longitudinal study

**DOI:** 10.7189/jogh.14.04127

**Published:** 2024-08-16

**Authors:** Ting Wang, Shouchuang Zhang, Qiaosheng Li, Haoran Liu, Shan Zhang, Weiyan Jian, Jing Guo

**Affiliations:** Department of Health Policy and Management, School of Public Health, Peking University, Beijing, China

## Abstract

**Background:**

The increasing prevalence of depressive symptoms has emerged as a critical public health issue globally, highlighting the need for analyses of the factors contributing to depressive symptoms within the Chinese population and the development of targeted recommendations for improving mental well-being. We aimed to explore the correlation between internet use and depressive symptoms and the role of socioeconomic inequalities in this association.

**Methods:**

We included data on 8019 residents aged 18 years and above, which we retrieved from the 2018 and 2020 waves of the China Family Panel Studies. We used latent profile analysis to categorise individuals’ internet usage patterns and multiple linear regression to determine their association with depressive symptoms.

**Results:**

Higher socioeconomic status (SES) was associated with fewer depressive symptoms (*τ* = −0.08; 95% confidence interval (CI) = −0.36, −0.18). Individuals in the high-dependence group presented a greater likelihood of developing depressive symptoms (*τ* = 0.04; 95% CI = 0.007, 0.66). We observed no significant difference in the interaction effect between individual-level SES and the four patterns of internet usage. However, compared with urban-dwelling respondents, those in rural areas had a stronger association between internet usage patterns and depressive symptoms, especially those in the high-dependence group (*τ* = −0.07; 95% CI = −1.47, −0.20).

**Conclusions:**

Our findings indicate a significant association between depressive symptoms and internet usage patterns, indicating a need for interventions related to internet use, especially those targeted at reducing the risk of depressive symptoms in individuals of lower SES.

In recent decades, depression has become more prevalent worldwide, emerging as one of the leading causes of disability and a major contributor to disease burdens [1]. Depressive symptoms affect approximately 350 million people globally and represent a significant public health concern [2,3], especially in middle- and low-income countries [4]. In the context of China, rapid economic growth and significant changes in the lifestyle of its population in recent decades have increased the risk of mental health issues, including depressive symptoms [5], with one study showing that depression and related symptoms contribute to 6.9% and 7.8% of total personal expected medical spending, respectively [6]. It is therefore necessary to examine the factors related to depressive symptoms in the Chinese population and to develop targeted recommendations to improve their mental health.

Studies have demonstrated an association between internet use and increased depressive symptoms [7–9], with several finding that internet usage in general and specific online activities such as social networking may be correlated with loneliness, low self-esteem, and depressive symptoms [10–12]. Moreover, most studies have shown that online activities are associated with reduced communication between family members, as well as increased symptoms of depression [13,14]; yet others also demonstrated that neither time spent on the internet nor network-related activities significantly affect the observed relationship between the level of internet usage and depressive symptoms [15,16]. Emerging evidence indicates that internet use may not play a significant role in the development of depressive symptoms, with one study noting that it may act as a protective factor against depressive symptoms [17]. This relationship between internet usage patterns and depressive symptoms deserves further study.

A significant correlation exists between socioeconomic status (SES), defined by factors such as educational level, income, and occupational status, and various health outcomes such as mortality, depression, and anxiety [18]. SES is typically conceptualised as an individual or group's social standing or class within the social hierarchy, especially at the family or community level, with less emphasis on the urban-rural distinctions [19–21]. Generally, individuals with low SES, characterised by lower education levels and poorer economic status, are more susceptible to life worries and depressive symptoms [22]. SES may also play a moderating role in the relationship between internet usage patterns and depressive symptoms. For example, the double jeopardy hypothesis suggests that individuals with lower SES often reside in disadvantaged areas, where their health outcomes are generally worse than for individuals in advantaged communities [23]. The relative deprivation hypothesis, in turn, posits that the health of individuals with lower SES may deteriorate if they reside in higher SES areas [24]. Consequently, it is possible that lower SES, combined with limited social support, could intensify the correlation between patterns of internet use and the manifestation of depressive symptoms [25]. To our knowledge, the association between SES and depressive symptoms has not been examined from a multilevel perspective, such as at the individual and regional levels.

In this study, we aimed to investigate the association between internet use and depressive symptoms by examining the moderating effect of SES (i.e. income and education level) within that association based on data from the China Family Panel Studies (CFPS).

## METHODS

### Data sources and sample composition

We retrieved data from the CFPS, a nationally representative longitudinal study conducted by the Institute of Social Science Survey of Peking University conducted biannually among 16 000 households within 25 of China's 31 provinces, including municipalities and autonomous regions [26]. The CFPS combined computer-assisted personal interviews and face-to-face interviews to collect the data [27]. Detailed information on its sampling and study procedures can be found elsewhere [28,29]. We used these data from the CFPS for our study. Specifically, we included individuals aged 18 years and above who had used the internet in 2018 and had reported depressive symptoms in both 2018 and 2020. We excluded participants if they lacked our variables of interest. After screening and matching using unique identification numbers (Figure S1 in the **Online Supplementary Document**), we obtained data on 8019 respondents who were fully surveyed in 2018 and 2020.

### Main outcome measures

#### Depressive symptoms

Radloff’s Center for Epidemiologic Studies Depression (CES-D-8) questionnaire, one of the most widely utilised self-assessment scales, has previously been used to assess respondents’ depressive symptoms [30]. It has been shown to have good reliability and validity in screening for depressive symptoms [31,32]. The CFPS 2018 contained a condensed version of the CES-D-8, aimed at reducing survey length and enhancing response rates (Table S1 in the **Online Supplementary Document**).

Our main outcome measures were depressive symptoms in 2018 and 2020. With responses scaling from 0 to 3 (0 = rarely, 1 = little, 2 = occasionally, and 3 = often) for each question, the respondents reported their depressive symptoms in the past week in the questionnaire. Negative questions were assigned a score of 0 to 3, and positive questions were assigned a score of 3 to 0. The CES-D-8 rates symptoms of depression on a scale of 0 to 24, where a higher score indicates worse symptoms. The scale’s internal consistency in our study, as measured by Cronbach’s alpha, was 0.754 for wave 2018 and 0.766 for wave 2020.

### Main measures of independent variables

#### Internet usage patterns

The CFPS 2018 questionnaire queried participants about the frequency of five internet usage items (i.e. study, work, social communication, entertainment, and business activities), with possible responses being ‘never’, ‘once every few months’, ‘once a month’, ‘2–3 times a month’, ‘1–2 times a week’, ‘3–4 times a week’, and ‘almost every day’. Here we graded these seven options on a scale of 1 to 7. Specifically, we assigned each option a corresponding value (i.e. 1 = never, 7 = almost every day), with higher values indicating greater frequencies of the five items of internet usage. These options show people's dependence on the internet in their studies, work and life. Based on pre-determined classificiation methods and relevant empirical research [33–35], we used the most typical features for each dimension. Among five internet use items, the frequency of using the internet for study and work served as the basis for primary classification, while the frequency of using the internet for social communication, entertainment, and business activities served as the reference for the secondary classification.

#### SES

Our second explanatory variable was the participants’ SES in 2018, as determined by a person’s level of education, income, and occupational prestige. Physical and mental health can be affected by SES through complex physiological and psychological mechanisms [36–40]. We used CFPS 2018 data on the participants’ level of education (1 = primary school, 2 = middle school, 3 = high school, 4 = college degree or above), self-evaluated income status (1 = low income, 2 = low-middle income, 3 = middle income, 4 = middle-high income, 5 = high income), and current employment status (0 = unemployed, 1 = employed) to determine their SES. In the context of China, however, other factors affect individuals’ social status, the first being political capital [41]. This factor, measured by membership in the Communist Party of China (CPC), was recently found to be a more accurate predictor of health than household income [42]. Following a prior study [43], we used the participants’ indication of whether any family member is a member of the Communist Party of China (0 = none, 1 = yes) as a measure of the variable of political capital at the household level. Based on these variables, we used principal component analysis to obtain a comprehensive SES index. To make all the fractions within the analysis positive, we added a constant of 3 to each fraction [37] to better identify the degree of SES.

The CFPS 2018 determined the participants’ residency area (urban or rural) by whether their address was located within a village or a residential committee community. In alignment with new standards set by the National Bureau of Statistics, the CFPS project team reassessed their urban or rural status based on their addresses at the family community level. The variable corresponding to this status in the new CFPS 2018 data set was denoted as ‘urban.’ In our study, we specifically labeled it as ‘urban2018,’ using it as an indicator of SES at the area level.

#### Potential confounders

We examined several possible confounders, including the following demographic and socioeconomic factors in 2018: region (West/Central/East/Northeast); sex (woman/man); marital status (married/unmarried); chronic diseases (yes/no); self-reported health status (bad/general/good); and age (18–30 years old/31–45 years old/over 45 years old). Previous studies have shown that depressive symptoms are associated with various demographic, socioeconomic, and health characteristics [28,29].

#### Statistical analysis

We used descriptive statistics to characterise our sample, presenting continuous variables as medians (MDs) and interquartile ranges (IQRs) and categorical variables as numbers (n) and percentages (%). We also conducted bivariate analyses where appropriate.

We classified individuals according to their patterns of response to episodic items [44], which we did by checking the distributions of different groups in the data and determining whether these distributions were meaningful. The variables observed in the application of latent profile analysis (LPA) are continuous [45]. Here we used Mplus, version 8.3 (Muthén & Muthén, Los Angeles, CA, USA) to check the number of unobserved categories (potential features of internet usage), describe their characteristics, and calculate the probability that each individual belongs to a given category. Generally, we used the Bayesian information criterion (BIC), sample-size adjustment (SABIC), the Akaike information criterion (AIC), and entropy for reference in LPA. Lower values of the AIC, BIC, and SABIC indicate a better fit for the model [46], while values of entropy higher than 0.8 are considered acceptable [47,48]. We further used the Lo-Mendell-Rubin (LMR) likelihood ratio test [49] and bootstrap likelihood ratio test (BLRT) [50] to compare different models, with their significance suggesting that the classification of a given model was better than another (*P* < 0.05) [51].

Lastly, we used multiple linear regression analysis to identify whether internet usage patterns were associated with depressive symptoms, and examined whether there was an interaction effect between the township of residence (compared with ‘Urban’ cases) and internet usage patterns on depressive symptoms.

We used Stata, version 15.0 (StataCorp LLC, College Station, TX, USA) for data analysis, with a *P* < 0.05 indicating statistical significance.

## RESULTS

### Descriptive statistical analysis

The respondents' average scores for depressive symptoms in 2018 and 2020 were 5.13 and 5.25, respectively. The median SES score of the overall sample was 2.69 (range: 0.82 to 8.89), indicating a generally low SES. More than half of the respondents were employed (n = 6688, 83.40%), married (n = 6881, 85.81%), not CPC members (n = 7898, 98.49%), had a middle income (n = 4369, 54.48%), and general health status (n = 4028, 50.23%). The respondents most frequently used internet for socialising and entertainment (MD = 7 (IQR = 6–7); MD = 6 (IQR = 5–7)) (**Table 1**).

**Table 1:** Descriptive statistics (n = 8019)*

Variables	
Depressive symptoms 2018, MD (IQR; range)	5 (2–7; 0, 24)
Depressive symptoms 2020, MD (IQR; range)	5 (2–8; 0, 24)
Internet use 2018, MD (IQR; range)	
*Study*	3 (1–6; 1, 7)
*Work*	1 (1–7; 1, 7)
*Social communication*	7 (6–7; 1, 7)
*Entertainment*	6 (5–7; 1, 7)
*Business activities*	4 (1–5; 1, 7)
SES, MD (IQR; range)	2.69 (2.38–3.53; 0.82, 8.89)
Working status	
*Unemployed*	1331 (16.60)
*Employed*	6688 (83.40)
Education	
*Primary school*	1141 (14.23)
*Middle school*	2956 (36.86)
*High school*	2968 (37.01)
*College degree or above*	954 (11.90)
Self-evaluated income status	
*Low*	713 (8.89)
*Low-middle*	1562 (19.48)
*Middle*	4369 (54.48)
*Middle-high*	1034 (12.89)
*High*	341 (4.25)
CPC member	
*None*	7898 (98.49)
*Yes*	121 (1.51)
Region	
*Northeast*	1115 (13.90)
*East*	3077 (38.37)
*Central*	1959 (24.43)
*West*	1868 (23.29)
Township	
*Urban*	5002 (62.38)
*Rural*	3017 (37.62)
Gender	
*Male*	3830 (47.76)
*Female*	4189 (52.24)
Marital status	
*Unmarried*	1138 (14.19)
*Married*	6881 (85.81)
Chronic diseases	
*None*	7070 (88.17)
*Yes*	949 (11.83)
Self-reported health status	
*Bad*	1517 (18.92)
*General*	4028 (50.23)
*Good*	2474 (30.85)
Age in years	
*18–30*	2466 (30.75)
*31–45*	3200 (39.91)
*>45*	2353 (29.34)

CPC – Communist Party of China, SES – socioeconomic status

*Values presented as n (%) unless specified otherwise.

### Model selection

Starting from the initial model, we built one to six profile models in a stepwise fashion. In the sixth model, the nonsignificant differences in the *P-*values of the LMR and BLRT suggested the superiority of the fifth model. The AIC, BIC, and SABIC were the lowest for the fifth model, the entropy was the best, and the LMR and BLRT were significant. However, the fifth model was deemed unsuitable, as one category represented only 2% of the total sample, falling short of the general LPA standard of at least 5% [33,52]. Considering this, we selected the fourth model for our analysis (**Table 2**). Using the internet to study and work frequently and having a high frequency of social, entertainment, and business activities indicates a heavy dependence on the internet, while utilising it for study, work, socialising, entertainment, and business activities was categorised as middle-high dependence. The low frequency of using the internet to study and work and the high frequency of its use for social, entertainment, and business activities indicated a middle dependence on the internet. Lastly, the low frequency of social, entertainment, and business activities indicated a low dependence on the internet. Following LPA, we categorised internet usage patterns into four levels: 1 for low dependence, 2 for middle dependence, 3 for middle-high dependence, and 4 for high dependence. According to the proportion of each category reported by the LPA, we obtained the proportion of respondents with different internet usage patterns: high dependence (36.3%), middle-high dependence (9.1%), middle dependence (45.2%), and low dependence (9.4%).

**Table 2:** Comparing models with different latent classes: fit indices (n = 8019)

								Proportion in class					
**Number**	**Log likelihood**	**AIC**	**BIC**	**SABIC**	**Entropy**	***P*-value for LMRT**	***P*-value for BLRT**	**1**	**2**	**3**	**4**	**5**	**6**
1	−88 050.362	176 120.723	176 190.619	176 158.841	-	-	-						
2	−79 907.756	159 847.511	159 959.344	159 908.499	0.990	<0.001	<0.001	0.44	0.56				
3	−77 162.039	154 368.079	154 521.849	154 451.938	0.982	<0.001	<0.001	0.44	0.47	0.09			
4	−73 439.854	146 935.708	147 131.416	147 042.437	0.991	<0.001	<0.001	0.36	0.45	0.09	0.09		
5	−71 969.790	144 007.579	144 245.225	144 137.179	0.994	<0.001	<0.001	0.35	0.45	0.09	0.09	0.02	
6	−71 201.119	142 482.239	142 761.822	142 634.709	0.993	0.931	1.000	0.05	0.09	0.09	0.30	0.45	0.02

AIC – Akaike information criterion, BIC – Bayesian information criterion, BLRT – bootstrapped likelihood ratio test, LMR – Lo-Mendell-Rubin test, SABIC – sample adjusted BIC

**Figure 1:**
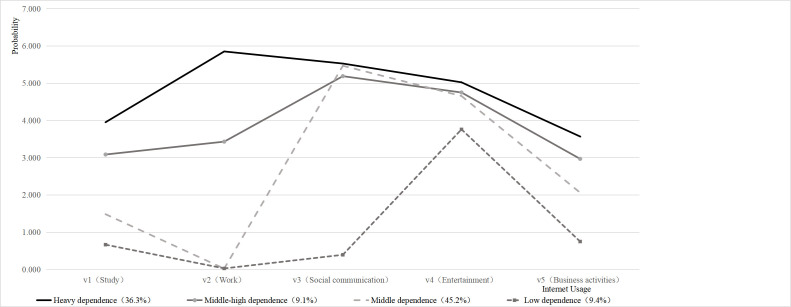
Internet usage patterns detected among internet users. They were determined by the estimated probability of respondents from each latent class answering from 1 to 7 of five items (i.e. study, work, social communication, entertainment, and business activities). The x-axis represents the five purposes for which the respondents use the internet: study, work, social communication, entertainment, and business activities. The y-axis denotes probability, with a range from 0 to 7. This probability value actually reflects the average response intensity or propensity for each category of respondents towards each question. Higher values indicate more frequent or intense behaviors related to the question within that category. The solid black line refers to the type with high dependence; the solid line with circular marks refers to the type with middle-high dependence; the dotted line refers to the type with middle dependence; and the dotted line with square marks refers to the type with low dependence.

We found that individuals with high dependence were most likely to use the internet for study or work, and that they showed the strongest dependence on social, entertainment, and business activities (**Figure 1**). Compared with high-dependence respondents, middle-high dependence ones simultaneously had significant dependence on using the internet to socialise, entertain, and conduct business activities. In contrast to those with high dependence and middle-high dependence, respondents with middle or low dependence on using the internet for study and work had an overall lower level of dependence. Unlike respodents with middle-high dependence, respondents with middle dependence were significantly more dependent on social communication. Moreover, with respect to dependence on social, entertainment, and business activities, the patterns of low dependence presented as the lowest by far.

### Bivariate and multiple linear regression analyses

We found that SES, region, township, gender, marital status, chronic diseases, self-reported health status, and age were related to respondents’ internet usage patterns (*P* < 0.05) (**Table 3**).

**Table 3:** Bivariate analysis of influencing factors of internet usage patterns (n = 8019)

	LD	MD	MHD	HD	*P*-value	DF	Cramer’s V
**SES**	-	-	-	-	<0.001	144	0.3686
**Region**					<0.001	9	0.0640
Northeast	166	523	63	363			
East	268	1,291	278	1,240			
Central	165	919	192	683			
West	150	897	196	625			
**Township**					<0.001	3	0.1776
Urban	338	1624	286	769			
Rural	411	2006	443	2142			
**Gender**					<0.001	3	0.1004
Male	441	1711	445	1592			
Female	308	1919	284	1319			
**Marital status**					<0.001	3	0.1962
Unmarried	29	330	129	650			
Married	720	3300	600	2261			
**Chronic diseases**					<0.001	3	0.0920
None	610	3155	643	2662			
Yes	139	475	86	249			
**Self-reported health status**					<0.001	6	0.0955
Bad	205	822	111	379			
General	336	1739	357	1596			
Good	208	1069	261	936			
**Age in years**					<0.001	6	0.2015
18–30	83	940	249	1194			
31–45	245	1404	306	1245			
>45	421	1286	174	472			

DF – degree of freedom, HD – high dependence, LD – low dependence, MD – middle dependence, MHD – middle-high dependence, SES – socioeconomic status

Controlling for confounding variables controlled, individual-level SES and internet usage patterns were significantly associated with depressive symptoms in 2018 and 2020. Specifically, higher SES was associated with fewer depressive symptoms (*τ* = −0.08; 95% confidence interval (CI) =  −0.36, −0.18) (**Table 4**). Compared with respondents in the low dependence group, those in the high dependence group (*τ* = 0.04; 95% CI = 0.007, 0.66) were more likely to experience depressive symptoms (Table S2 in the **Online Supplementary Document**).

**Table 4:** Multiple linear regression of the relationships among SES, internet usage patterns, and depressive symptoms (n = 8019)

	Depressive symptoms 2018		Depressive symptoms 2020	
**Variables**	***τ* (95%CI)**	***P*-value**	***τ(*95%CI)**	***P*-value**
SES	−0.07 (−0.35, −0.17)	<0.001	−0.08 (−0.36, −0.18)	<0.001
Internet usage patterns				
*LD*	ref		ref	
*MD*	−0.003 (−0.30, 0.25)	0.863	−0.005 (−0.34, 0.27)	0.817
*MHD*	0.04 (0.16, 0.88)	<0.01	0.01 (−0.23, 0.44)	0.723
*HD*	0.06 (0.17, 0.80)	<0.01	0.04 (0.007, 0.66)	<0.05
Region				
*East*	ref		ref	
*Northeast*	0.005 (−0.19, 0.29)	0.672	0.02 (−0.06, 0.44)	0.141
*Central*	0.04 (0.12, 0.48)	<0.01	0.04 (0.17, 0.57)	<0.001
*West*	0.08 (0.45, 0.83)	<0.001	0.09 (0.61, 1.04)	<0.001
Township				
*Urban*	ref	0.672	ref	0.141
*Rural*	0.06 (0.25, 0.56)	<0.001	0.05 (0.22, 0.56)	<0.001
Gender				
*Female*	ref		ref	
*Male*	−0.06 (−0.56, −0.27)	<0.001	−0.06 (−0.58, −0.25)	<0.001
Marital status				
*Unmarried*	ref		ref	
*Married*	−0.07 (−0.96, −0.45)	<0.001	−0.06 (−0.95, −0.41)	<0.001
Chronic diseases				
*None*	ref		ref	
*Yes*	0.04 (0.12, 0.64)	<0.01	0.04 (0.15, 0.70)	<0.01
Self-reported health status				
*Bad*	ref		ref	
*General*	−0.24 (−1.87, −1.42)	<0.001	−0.16 (−1.45, −0.96)	<0.001
*Good*	−0.37 (−3.01, −2.54)	<0.001	−0.25 (−2.33, −1.81)	<0.001
Age				
*18*–30	ref		ref	
*31*–*45*	0.01 (−0.12, 0.27)	0.453	−0.02 (−0.33, 0.09)	0.265
*>45*	−0.10 (−0.96, −0.52)	<0.001	−0.10 (−1.07, −0.58)	<0.001
*con*	(7.59, 8.54)	<0.001	(6.34, 7.24)	<0.001

CI – confidence interval, con – constant, HD – high dependence, LD – low dependence, MD – middle dependence, MHD – middle-high dependence, ref – reference, SES – socioeconomic status

Lastly, we saw no significant difference in the interaction effect of individual-level SES and the four internet usage patterns, while the interaction at the urban-rural level was significant. Compared with urban residents, rural respondents showed a stronger correlation between internet usage patterns and depressive symptoms, especially within the high dependence group (*τ* = −0.07; 95% CI = −1.47, −0.20) (**Table 5**).

**Table 5:** Results of multiple linear regression with interaction (n = 8019)

	Model 1		Model 2	
	***τ* (95%CI)**	***P*-value**	***τ* (95%CI)**	***P*-value**
**Depressive symptoms 2018**				
SES	−0.04 (−0.47, 0.19)	0.399	−0.08 (−0.36, −0.18)	<0.001
Internet usage patterns				
*LD*	ref		ref	
*MD*	0.03 (−0.75, 1.15)	0.685	0.01 (−0.28, 0.46)	0.639
*MHD*	0.12 (0.11, 2.82)	<0.05	0.07 (0.36, 1.31)	<0.01
*HD*	0.07 (−0.50, 1.50)	0.326	0.06 (0.05, 0.83)	<0.05
Interaction				
*SES × LD*	ref		ref	
*SES × MD*	−0.04 (−0.45, 0.27)	0.624	-	
*SES × MHD*	−0.09 (−0.77, 0.13)	0.160	-	
*SES × HD*	−0.06 (−0.48, 0.23)	0.498	-	
Interaction				
*Rural × LD*	ref		ref	
*Rural × MD*	-		−0.03 (−0.79, 0.31)	0.390
*Rural × MHD*	-		−0.04 (−1.43, −0.02)	<0.05
*Rural × HD*	-		−0.05 (−1.19, −0.05)	<0.05
Township				
*Urban*	ref		ref	
*Rural*	0.06 (0.24, 0.55)	<0.001	0.11 (0.28, 1.29)	<0.01
*Covariates*				
*con*	(6.87, 8.72)	<0.001	(7.40, 8.45)	<0.001
**Depressive symptoms 2020**				
SES	−0.06 (−0.56, 0.14)	0.236	−0.08 (−0.40, −0.22)	<0.001
Internet usage patterns				
*LD*	ref		ref	
*MD*	0.03 (−0.76, 1.22)	0.646	0.03 (−0.21, 0.59)	0.351
*MHD*	0.001 (−1.54, 1.56)	0.987	0.03 (−0.09, 0.88)	0.113
*HD*	0.05 (−0.69, 1.41)	0.501	0.06 (0.04, 0.87)	<0.05
Interaction				
*SES × LD*	ref		ref	
*SES × MD*	−0.04 (−0.48, 0.27)	0.580	-	
*SES × MHD*	−0.004 (−0.52, 0.52)	0.995	-	
*SES × HD*	−0.05 (−0.47, 0.28)	0.614	-	
Interaction			-	
*Rural × LD*	ref		ref	
*Rural × MD*	-		−0.05 (−1.11, 0.13)	0.121
*Rural × MHD*	-		−0.04 (−1.48, 0.05)	0.067
*Rural × HD*	-		−0.07 (−1.47, −0.20)	<0.05
Township				
*Urban*	ref		ref	
*Rural*	0.05 (0.22, 0.56)	<0.001	0.12 (0.39, 1.53)	<0.01
*Covariates*				
*con*	(6.76, 8.70)	<0.001	−0.08 (7.15, 8.27)	<0.001

CI – confidence interval, con – constant, HD – high dependence, LD – low dependence, MD – middle dependence, MHD – middle-high dependence, ref – reference, SES – socioeconomic status

## DISCUSSION

Based on data from the CFPS, we investigated depressive symptoms among Chinese adults, examined the relationship between their internet usage patterns and depressive symptoms, and the interaction effect of urban-rural differentials and SES on depressive symptoms. We found that internet usage patterns are significantly associated with depressive symptoms both in the short term and long term, particularly in the high-dependence group. We also observed that SES might serve as a moderating factor in the relationship between internet usage patterns and depressive symptoms.

These findings align with those of most prior studies which have shown that internet use is associated with depressive symptoms. We further saw a heightened risk of depressive symptoms among individuals with high internet dependency, which also agrees with prior research [53]. Excessive computer-mediated communication and highly internet-dependent behavior that reduce the available time for conventional face-to-face interaction might be among the reasons for the increase in depressive symptoms [54]. For example, Tanis et al. [55] reported an increase in the frequency of self-diagnosis due to internet usage, which may lead to an increase in self-reported depressive symptoms, especially in young adults [56]. According to Banjanin et al. [11]., the true reasons for the relationship between internet use and depressive symptoms may be related to other aspects of internet use, such as email checking, general net browsing, and computer games. Further research could deepen the understanding of these issues, especially with respect to the patterns of internet use for specific purposes.

An especially significant finding from our study is that SES is associated with depressive symptoms, as it was previously found to play a key role in health-related issues [57]. Previous studies have consistently shown that a higher level of SES is associated with better health status [58–60], which is in line with our findings. For example, SES may directly impact depressive symptoms; recent studies have shown that people with lower SES are more likely to reside in disadvantageous environments, and are therefore more likely to have negative emotions such as loss and depressive symptoms [61]. Moreover, according to the social causation hypothesis, low SES tends to lead to life stresses, which may result in detrimental effects for mental health [62], while high SES suggest that an individual has a sufficiently high income to enjoy better material living and broader interests, potentially enabling them to mitigate depressive symptoms by allowing them to engage in various activities [63]. Conversely, SES can indirectly affect depressive symptoms through lifestyle-related factors. Here, the health risk behavior theory suggests that individuals with low SES more often have poor health, primarily due to their own behavior, while their behavior and lifestyle themselves are influenced by SES in significant ways [64]. Relatedly, individuals in lower socioeconomic groups are more likely to engage in health risk behaviors [59]. Aimee et al. [65] found that individuals with lower SES are more prone to psychological distress than those with higher SES, so strategies to reduce depressive symptom should be directed towards Chinese adults with low SES.

One of our key observations is that area-level SES modulates the relationship between patterns of internet usage and depressive symptoms, which aligns with the results of previous studies [66]. A plausible explanation is that individuals residing in regions with lower SES, constrained by their limited social resources and lessened social support, could exacerbate the association between internet use and depressive symptoms [67,68]. Consistent with the double jeopardy hypothesis, individuals with lower SES in such areas experience a compounding disadvantage with respect to social resources and interpersonal relationships [69]. Specifically, employment status affects an individual's economic stability, social position, and level of stress in daily life. Job insecurity or unemployment, in turn, can increase psychological stress, consequently impacting mental health [70]. In this sense, educational level are also closely linked to cognitive ability, health consciousness, and the capacity to access resources, with higher educational attainment often correlating with improved health outcomes and a reduced risk of depressive symptoms [71]. Income level directly influences quality of life and the ability to obtain medical and social support, with financial hardship recognised as a significant factor contributing to psychological stress and depressive symptoms [72]. Lastly, membership in the CPC was found to correlate with higher social standing, easier access to resources, and stronger social networks; such associations can offer supplementary social and economic support, which might contribute to the easing of depressive symptoms [42]. Consequently, these individuals may encounter higher levels of social stressors and challenges, potentially intensifying the association between internet use and depressive symptoms [23]. Moreover, the health behavior hypothesis suggests that residents in lower SES regions may engage in detrimental internet behaviors, such as prolonged usage or online gaming, which potentially suggests an increased risk for the manifestation of depressive symptoms [24,73]. Notably, SES and depressive symptoms had a weak association, so more in-depth studies are needed to establish a concrete relationship between the two.

### Strengths and limitations

This study, based on nationally representative data from China, facilitates cross-country comparisons and adds to the knowledge on the effects of SES and internet use on depressive symptoms. To our knowledge, it is one of the first to empirically determine the interaction of individual-level and area-level SES within the association between patterns of internet usage and depressive symptoms from a multilevel perspective in mainland China. However, this study has several limitations that should be acknowledged. First, further studies are needed on the mechanisms of patterns of internet use and depressive symptoms. Second, we did not manage to account for all potential confounding variables, such as family structure factors or objective measures of physical health. Specifically, self-reported health status inherently contains a degree of subjectivity and may not objectively reflect the actual health status of an individual. Furthermore, owing to inherent limitations in the data, we could not access relevant indicators involving the duration participants spend on each type of internet activity, thereby constraining further investigative analysis. This should be the focus or should be considered in subsequent studies. Finally, the CES-D serves not as a clinical diagnostic instrument but rather as a screening tool and a self-report instrument, which might lead to over- or under-reporting of depressive symptoms. Therefore, a primary focus should be on directing attention towards individuals residing in lower SES areas and offering enhanced social support, alongside implementing internet use training programmes to guide them towards fostering positive and healthy internet habits.

## CONCLUSIONS

Our findings indicate a significant association between internet usage patterns and depressive symptoms, and suggest that SES may function as a moderator in the relationship between internet use and depressive symptoms. Considering this, policymakers and stahekolders should develop and implement interventions for internet use, focussing on the reduction of depression symptom risk in individuals with lower SES. Such interventions should specifically target individuals in lower SES areas who should receive comprehensive guidance on internet use adapted to their circumstances.

## Additional material


Online Supplementary Document
