# Supplementary material for: Socioeconomic inequalities in the relationship between internet usage patterns and depressive symptoms: Evidence from a Chinese longitudinal study
Source: J Glob Health. 2024 Aug 16;14:04127. doi: 10.7189/jogh.14.04127 (PMC11327895; doi:10.7189/jogh.14.04127)
Supplement: Online Supplementary Document [file jogh-14-04127-s001.pdf]

# Supplemental figure

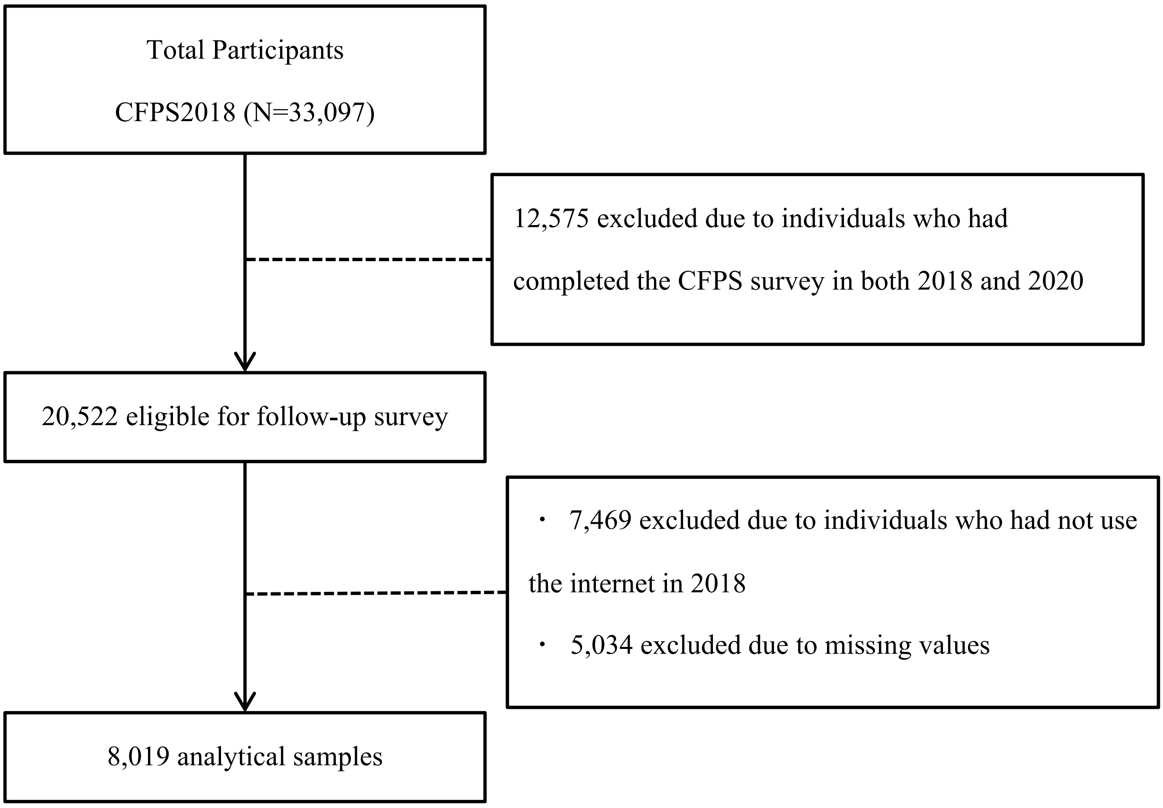


Figure S1. Data cleaning process

## Supplemental tables

Table S1. CES-D-8 scale

| Variable | Item |
|---|---|
| QN406（CES-D6） | I felt depressed |
| QN407（CES-D7） | I felt everything I did was an effort |
| QN411（CES-D11） | My sleep was restless |
| QN412（CES-D12） | I was happy |
| QN414（CES-D14） | I felt lonely |
| QN416（CES-D16） | I enjoyed life |
| QN418（CES-D18） | I felt sad |
| QN420（CES-D20） | I could not 'get going' |

Table S2. Results of multiple linear regression between Internet use and depressive symptoms within four different groups

| Variables | 2018(Depressive Symptoms) | | 2020 (Depressive Symptoms) | |
|---|---|---|---|---|
| | Beta | 95%CI | Beta | 95%CI |
| **LD Group** | | | | |
| **SES** | -0.03 | (-0.47, 0.21) | -0.05 | (-0.64, 0.08) |
| **Internet Use** | | | | |
| Study | -0.04 | (-0.25, 0.06) | -0.03 | (-0.23, 0.09) |
| Work | 0.007 | (-1.14, 1.39) | 0.09 | (-0.24, 3.57) |
| Social Communication | -0.02 | (-0.40, 0.21) | -0.06 * | (-0.64, -0.02) |
| Entertainment | -0.05 | (-0.17, 0.04) | -0.009 | (-0.13, 0.10) |
| Business Activities | 0.01 | (-0.17, 0.24) | 0.04 | (-0.64, 0.08) |
| **Covariates** | | | | |
| **Con.** | -- | (6.54, 11.57) | -- | (3.07, 8.85) |
| **MD Group** | | | | |
| **SES** | -0.06 ** | (-0.40, -0.10) | -0.07 *** | (-0.48, -0.18) |
| **Internet Use** | | | | |
| Study | -0.03 | (-0.10, 0.005) | -0.03 | (-0.10, 0.005) |
| Work | -0.03 * | (0.03, 1.51) | 0.02 | (-0.21, 1.34) |
| Social Communication | -0.03 | (-0.26, 0.005) | -0.01 | (-0.20, 0.10) |
| Entertainment | -0.01 | (-0.09, 0.12) | 0.005 | (-0.06, 0.07) |
| Business Activities | 0.03 | (-0.001, 0.12) | 0.001 | (-0.06, 0.07) |
| **Covariates** | | | | |
| **Con.** | -- | (7.21, 9.91) | -- | (6.84, 9.78) |
| **MHD Group** | | | | |
| **SES** | -0.11 ** | (-0.83, -0.17) | -0.05 | (-0.63, 0.17) |
| **Internet Use** | | | | |
| Study | -0.06 | (-0.24, 0.03) | -0.02 | (-0.17, 0.11) |
| Work | 0.01 | (-0.28, 0.40) | 0.02 | (-0.23, 0.44) |
| Social Communication | -0.007 | (-0.22, 0.18) | -0.006 | (-0.19, 0.16) |
| Entertainment | 0.08 * | (0.01, 0.35) | 0.05 | (-0.06, 0.26) |
| Business Activities | 0.03 | (-0.08, 0.20) | 0.05 | (-0.05, 0.24) |
| **Covariates** | | | | |
| **Con.** | -- | (5.17, 9.93) | -- | (3.50, 8.59) |
| **HD Group** | | | | |
| **SES** | -0.08 *** | (-0.39, -0.14) | -0.08 *** | (-0.45, -0.18) |
| **Internet Use** | | | | |
| Study | -0.02 | (-0.09, 0.02) | -0.01 | (-0.08, 0.40) |
| Work | -0.02 | (-0.56, 0.10) | -0.02 | (-0.52, 0.21) |
| Social Communication | -0.01 | (-0.16, 0.09) | -0.01 | (-0.15, 0.08) |
| Entertainment | 0.006 | (-0.07, 0.10) | -0.01 | (-0.13, 0.06) |
| Business Activities | 0.06 ** | (0.03, 0.17) | 0.07 ** | (0.06, 0.21) |

| | | | | |
|---|---|---|---|---|
| **Covariates** | | | | |
| **Con.** | -- | (6.99, 11.76) | -- | (6.21, 11.51) |

Note. SES, Socioeconomic Status; HD, heavy dependence; MHD, middle-high dependence; MD, middle dependence; LD, low dependence. CI, confidence interval, *$P$<0.05 **$P$<0.01 ***$P$<0.001.
